# Chrysophanol Regulates Cell Death, Metastasis, and Reactive Oxygen Species Production in Oral Cancer Cell Lines

**DOI:** 10.1155/2020/5867064

**Published:** 2020-05-26

**Authors:** Po-Chih Hsu, Ching-Feng Cheng, Po-Chun Hsieh, Yi-Hsuan Chen, Chan-Yen Kuo, Huey-Kang Sytwu

**Affiliations:** ^1^Graduate Institute of Medical Sciences, National Defense Medical Center, Taipei, Taiwan; ^2^Department of Dentistry, Taipei Tzu Chi Hospital, Buddhist Tzu Chi Medical Foundation, New Taipei City, Taiwan; ^3^Department of Pediatrics, Taipei Tzu Chi Hospital, Buddhist Tzu Chi Medical Foundation, New Taipei City, Taiwan; ^4^Institute of Biomedical Sciences, Academia Sinica, Taipei, Taiwan; ^5^Department of Pediatrics, Tzu Chi University, Hualien, Taiwan; ^6^Department of Chinese Medicine, Taipei Tzu Chi Hospital, Buddhist Tzu Chi Medical Foundation, New Taipei City, Taiwan; ^7^Department of Research, Taipei Tzu Chi Hospital, Buddhist Tzu Chi Medical Foundation, New Taipei City, Taiwan; ^8^National Institute of Infectious Diseases and Vaccinology, National Health Research Institutes, Zhunan, Miaoli County, Taiwan, China; ^9^Department of Microbiology and Immunology, National Defense Medical Center, Taipei, Taiwan, China

## Abstract

**Background:**

Oral cancer belongs to the class of head and neck cancers and can be life threatening if not diagnosed and treated early. Activation of cell death via apoptosis or reactive oxygen species (ROS) accumulation and inhibition of cell cycle progression, migration, and epithelial-to-mesenchymal transition (EMT) may be a good strategy to arrest the development of oral cancer. In this study, we analyzed the possible action of chrysophanol isolated from the rhizomes of *Rheum palmatum* on the oral cancer cell lines FaDu (human pharynx squamous cell carcinoma) and SAS (human tongue squamous carcinoma) by investigating whether chrysophanol could influence cell death.

**Method:**

Cell viability was measured by using the MTT assay. For the detection of apoptosis, terminal deoxynucleotidyl transferase dUTP nick-end labeling (TUNEL) staining and subG1 population analysis were used. We also examined cell cycle progression and ROS levels by flow cytometry. Additionally, the expression of p53, p21, procaspase 3, cyclin D1, CDK4, cdc2, CDK2, E-cadherin, vimentin, and PCNA was evaluated by western blotting.

**Conclusion:**

Chrysophanol has an anticancer effect on FaDu and SAS cell lines. There is an increase in subG1 accumulation, ROS production, and cell cycle G1 arrest after treatment with chrysophanol. On the other hand, chrysophanol inhibited cell migration/metastasis and EMT. We proposed that chrysophanol may be a good candidate compound on oral cancer treatment in the further.

## 1. Introduction

Recently, global cancer statistics sourced from GLOBOCAN 2018 estimating the incidence and mortality of 36 cancers in 185 countries revealed that the incidence of Head and Neck Squamous Cell Carcinoma (HNSCC) was 354.9 thousand new cases with a mortality rate of approximately 50% [1]. In comparison, statistics gathered in 2015 estimated the incidence to be 300 thousands new cases with 48% mortality [2], indicating that both incidence and mortality rate are still rising. HNSCC is derived from a mutation or abnormality in the squamous cell lining of the oral cavity, oropharynx, larynx, or hypopharynx [3], and factors including alcoholic consumption [4], smoking [5], and human papillomavirus (HPV) infection in nonsmokers [6] are known to put people at risk. Tumor recurrence and metastasis of HNSCC may lead to a poor prognosis [7].

The development and progression of HNSCC correlates with cells having several characteristic including limitless replicative potential, genetic instability, metabolic change, self-sufficiency in growth signals, insensitivity to antigrowth signals, ability to avoid cell death, angiogenesis initiation, and ability to invade and metastasize [4, 8, 9]. However, HNSCC are diagnosed on the basis of TNM staging systems, and clinical treatment modalities, including surgery, chemotherapy, radiotherapy, and developing immunotherapy, are then applied [10]. Since homogeneous treatments based on the TNM staging for different HNSCC tumors in clinical practice, the high mortality rate of the patients is still a limitation [11]. At present, improved technologies and refined algorithms are available to analyze databases of HNSCC profiles, predict cells that may undergo metastasis, and detect tumors in a single cell, revealing factors that correlate with tumor progression and metastasis. On the other hand, it is critical to discover candidate compounds to alleviate carcinogenesis.

Recently, botanical components have been reported as adjuvant therapies in anticancer treatment. Chrysophanol (1,8-dihydroxy-3-methyl-anthraquinone), a secondary metabolite extract of rhubarb (*Rheum undulatum L.*) [12], has been demonstrated in both European and Chinese Pharmacopoeia [13]. Belonging to anthraquinone family [6], it is a derivative of the 9,10 anthracendione ring with a 1,8-dihydroxy-3-methyl group. Members of the anthraquinone family are used as anticancer drugs and anthracyclines in clinical practice [14]. Accumulating evidence shows that chrysophanol has multiple roles as an anticancer, antioxidant, anti-inflammatory, antineoplastic, antiarthritic, antifungal, antibacterial, antiviral, antipathogenic, antidiabetic, neuroscientist-protective, antiplatelet, antiaging, and antisclerosis agent [15, 16]. Additionally, it has been reported that chrysophanol alleviates the development of cancer via epithelial-to-mesenchymal transition (EMT) [17, 18]; however, the pharmacological effect of chrysophanol on HNSCC is still uncertain.

This study investigated the pharmacological effects and mechanisms of chrysophanol in HNSCC cell lines, including SAS and FaDu. The results showed that chrysophanol prevents carcinogenesis by decreasing cell proliferation and metastasis, as well as increasing cell apoptosis. We propose that chrysophanol may be a good candidate compound for HNSCC treatment in the future.

## 2. Materials and Methods

### 2.1. Reagents

Chrysophanol and *N*-acetyl-L-cysteine were provided by Cayman Chemical (MI, USA). Cisplatin was purchased from Sigma (MO, USA). Dulbecco's Modified Eagle Medium, CellROX® Oxidative Stress Reagents, and Pierce BCA Protein Assay kit were provided by ThermoFisher Scientific (MA, USA). Fetal bovine serum was purchased from Corning (NY, USA). Penicillin/streptomycin was provided from Bioindustry (London). PhosSTOP and complete ULTRA tablets, the In Situ Cell Death Detection kit, and fluorescein were purchased from Roche (Germany). Z-VAD-FMK was provided from R&D systems (MN, USA). Anti-cyclin A2, E-cadherin, cyclin E1, cyclin D1, CDK4, cdc2, and CDK2 antibodies were purchased from Cell Signaling (MA, USA). The anti-EpCAM antibody and Apoptosis/Necrosis Assay kit were purchased from Abcam (UK). Anti-vimentin and PCNA were provided from BioLegend (CA, USA) and ABclonal (MA, USA), respectively. Anti-*β* actin antibody was purchased from Santa Cruz Biotechnology (TX, USA).

### 2.2. Cell Culture

FaDu (human pharynx squamous cell carcinoma) and SAS (human tongue squamous carcinoma) cell lines were obtained from ATCC and National Defense Medical Center, respectively. Cells were analyzed for mycoplasma and tested negative. Cell lines were cultured in Dulbecco's Modified Eagle Medium containing 10% fetal bovine serum and 1% penicillin/streptomycin and incubated in a 5% CO_2_ atmosphere at 37°C.

### 2.3. Cell Viability and Cytotoxicity Assay and IC50 Value Determination

Cell viability and cytotoxicity were assessed by using the MTT assay. Cells (1 × 10^6^) were seeded in 96-well plates with the indicated concentrations of chrysophanol and then treated for 24 hours. To the cells was added 3-(4,5-dimethylthiazol-2-yl)-2,5-diphenyltetrazolium bromide (MTT, ThermoFisher Scientific, MA, USA) and incubated at 37°C for 2 hours. Finally, formazan was solubilized with DMSO. The concentration was determined from the optical density at 570 nm. Absorbance was measured with a TECAN infinite M200 PRO (Switzerland). According to a previous study [19], measurements were performed, and the concentration required for 50% inhibition of viability (IC50) was determined graphically from a standard curve drawn by plotting the log of the drug concentration on the *X* axis and % cell growth inhibition or % cytotoxicity on the *Y* axis. Additionally, the IC50 values were estimated as the drug concentration at the 50% position on the *Y* axis. The relationship should be sigmoidal, with the log of the drug concentration on the *X* axis and “response/measurement” on the *Y* axis. The Prism website has some good guidelines for this. We used GraphPad Prism8 software to determine the IC50 value. Moreover, IC50 values were calculated using the nonlinear regression program origin. The average of two measurements (in a duplicate manner) was taken in determination. The IC50 value was derived using curve fitting methods with GraphPad Prism8 statistical software.

### 2.4. Protein Extraction and Western Blotting

Protein was extracted from cells and subjected to western blotting according to our previous study [19]. The intensities of the reactive bands were analyzed by using Image J.

### 2.5. Measurement of Intracellular ROS Generation

The intracellular ROS levels were examined with CellROX® Oxidative Stress Reagents, according to the manufacturer's instructions. The relative level of ROS was analyzed by using a Coulter Cytomic FC 500 (Beckman, CA, USA).

### 2.6. Analysis of the Cell Cycle

Cells were treated with chrysophanol at the indicated times before harvesting and fixing in ice-cold 70% ethanol for 1 h. Cells were then washed with PBS and incubated in propidium iodide staining buffer (50 *μ*g/ml propidium iodide, 0.1 mg/ml DNase-free RNase A, and 0.5% Triton X-100) for 30 min at 37°C in the dark. The DNA content was analyzed by flow cytometry (Beckman Coulter Cytomic FC 500, CA, USA).

### 2.7. Apoptosis Staining

Apoptosis-associated DNA fragmentation was visualized by using the terminal deoxyribonucleotidyl transferase-mediated dUTP-digoxigenin nick-end labeling (TUNEL) apoptosis detection kit (Roche), according to the manufacturer's instructions. The cells were finally counterstained with DAPI and analyzed by the Leica DM6000 Microscope (Germany).

### 2.8. Wound Healing Assay

Cells were plated at an initial density of 1 × 10^5^ cells/ml to form a monolayer. During the last 4 h, cytosine*β*-D-arabinofuranoside (Sigma) was added at a final concentration of 4 *μ*m to prevent cell proliferation. Cells were washed twice with PBS and incubated with medium containing serum at various concentrations (0, 30, and 70 *μ*m) of chrysophanol for 0, 4, 8, and 12 h. Then, cells were wounded by scraping with a pipette tip to make an approximately 400-*μ*m gap in the cell monolayer. Cell migration was observed at different time intervals and photographed at 3 marked locations on each dish using a phase-contrast microscope. The number of migrated cells was counted and averaged. All experiments were carried out in triplicate and repeated at least 3 times.

### 2.9. Statistical Analysis

Data were expressed as the mean ± standard error of the mean (SEM), and statistical comparisons were calculated by one-way or two-way analysis of variance (ANOVA) followed by a Bonferroni post hoc test. A value of *p* < 0.05 was considered to indicate the statistical significance.

## 3. Results and Discussion

### 3.1. Chrysophanol Causes Cell Death in HNSCC

**Figure 1:**
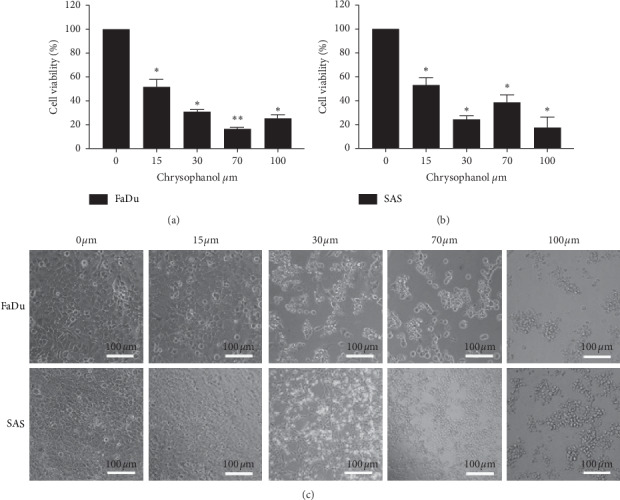
Chrysophanol causes cell death in the HNSCC cell lines FaDu and SAS. (a and b) After the incubation time course, cell viability was determined by using the MTT assay. (c) Morphological changes in cells were observed at the indicated concentrations. Bar = 100 *μ*m. All data are presented as mean ± SEM, *n* = 3. ^*∗*^*p* < 0.05. ^*∗∗*^*p* < 0.01 compared with control (0 *μ*m).

**Figure 2:**
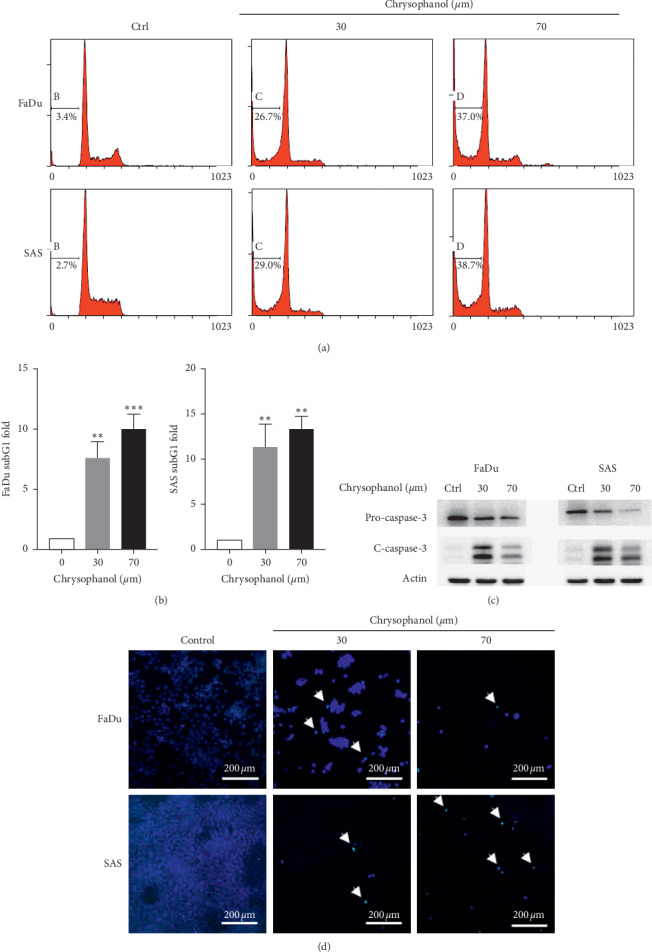
Chrysophanol induced apoptosis in FaDu and SAS. (a) Cell death via apoptosis was described by the SubG1 population of FaDu and SAS in the absence (control) and presence of chrysophanol (30 and 70 *μ*m). (b) The quantification in fold-changes of the SubG1 population. (c) The expressions of procaspase 3 and cleaved-caspase 3 (c-caspase-3) were analyzed by western blotting. (d) TUNEL staining showing the apoptotic phenomena (arrow) induced by chrysophanol in FaDu and SAS cell lines. Bar = 200 *μ*m. All data are presented as mean ± SEM, *n* = 3. ^*∗∗*^*p* < 0.01. ^*∗∗∗*^*p* < 0.001 compared with control (Ctrl or 0 *μ*m).

To investigate the cytotoxic effect of chrysophanol on HNSCC, the cell viability of FaDu and SAS was analyzed after treatment with a concentration series of chrysophanol (0, 15, 30, 70, and 100 *μ*m) for 24 hours (Figure 1). The IC50 value at 24 h was 9.64 ± 1.33 *μ*m and 12.60 ± 2.13 *μ*m in FaDu and SAS, respectively. Results showed that chrysophanol (15–100 *μ*m) inhibited cell viability in a concentration-dependent manner (Figures 1(a) and 1(b)). To characterize chrysophanol-induced cell death, the changes in cellular morphology were determined. Phase-contrast microscopy showed that cells treated with chrysophanol (30, 60, and 100 *μ*m) for 24 h underwent marked apoptotic changes, including the formation of membrane blebs and apoptotic bodies (Figure 1(c)). Interestingly, cell viability was decreased dramatically after treatment with 30 *μ*m chrysophanol and reached the threshold at 100 *μ*m (Figure 1). To further determine the apoptotic effect of chrysophanol on FaDu and SAS, the subG1 population and TUNEL were analyzed (Figure 2).

### 3.2. Chrysophanol Causes Apoptosis in HNSCC

It is well known that the process of programmed cell death, or apoptosis, is generally characterized by distinct methods, including morphological changes, subG1 population analysis, caspase-3 expression analysis, and TUNEL staining [20]. Results showed that chrysophanol (30 and 70 *μ*m) caused an increase in the subG1 phase in FaDu and SAS (Figures 2(a) and 2(b)), which is an indicator of apoptosis [21]. Additionally, a decrease in expression of procaspase 3 was detected in chrysophanol (30 and 70 *μ*m) treated FaDu and SAS (Figure 2(c) and supplementary Figure 1), similar to the previous findings in breast and hepatic cancer cell lines [22, 23]. DNA fragmentation was also observed in the presence of chrysophanol (30 and 70 *μ*m) treatment (Figure 2(d)). Taken together, these findings suggest that chrysophanol triggers FaDu and SAS to undergo apoptosis in a dose-dependent manner.

### 3.3. Influence of Chrysophanol on ROS Production and Cell Cycle Regulation

**Figure 3:**
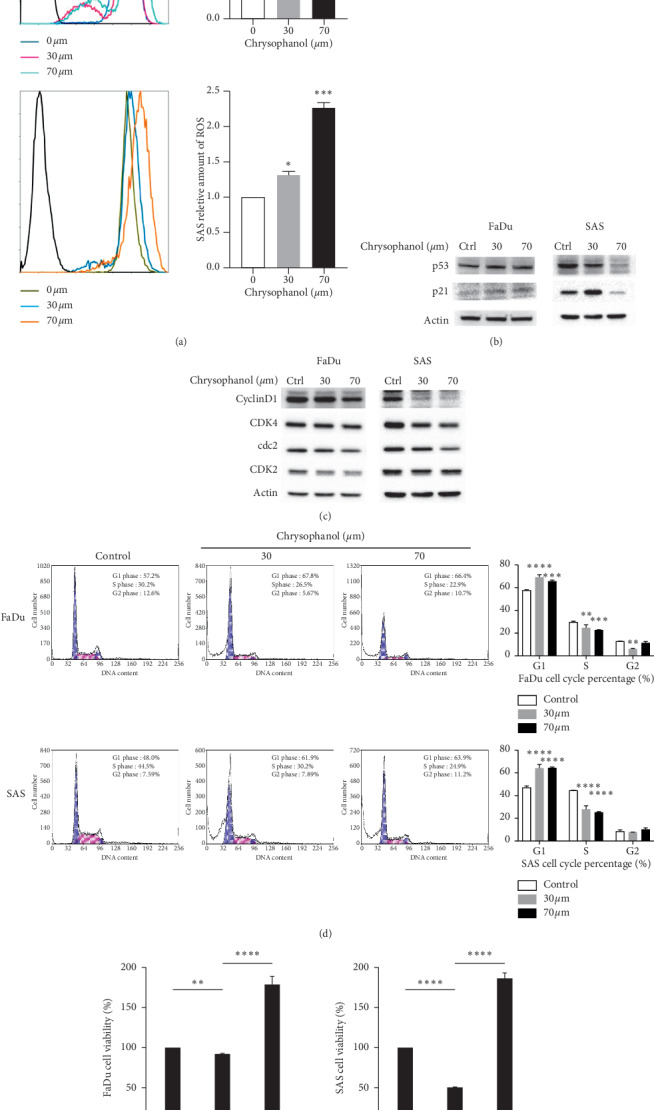
Chrysophanol regulates ROS production and the expression of p53, p21, cyclinD1, CDK4, cdc2, CDK2, and cell cycle progression. (a) The level of intracellular ROS determined by using the CellROX® oxidative stress reagents in chrysophanol-treated cells (30 and 70 *μ*m) or control (Ctrl, 0 *μ*m), and the fluorescence detected by flow cytometry. (b, c) ROS generation expressed as the mean fluorescence intensity. The expressions of (b) p53, p21, (c) cyclinD1, CDK4, cdc2, and CDK2 analyzed by western blotting in chrysophanol-treated cells (30 and 70 *μ*m) or control (Ctrl, 0 *μ*m). (d) DNA distribution histogram of chrysophanol-treated cells (30 and 70 *μ*m) or control (0 *μ*m). (e) Effect of NAC on chrysophanol (70 *μ*m) treated cells. Cell viability was determined by using the MTT assay in chrysophanol-treated cells in the absence (70 *μ*m) or presence (70 *μ*m + NAC) of 20 mm NAC and in the control (0 *μ*m). All data are presented as mean ± SEM, *n* = 3. ^*∗∗*^*p* < 0.01. ^*∗∗∗*^*p* < 0.001. ^*∗∗∗∗*^*p* < 0.0001 compared with control or 0 *μ*m.

Kim et al. demonstrated that p53-p21-regulated cancer cell invasion and apoptosis are involved in reactive oxygen species (ROS) production [24]. In this study, to further determine the pharmacological mechanism of chrysophanol acting on FaDu and SAS, the level of ROS and the expression of p53 and p21 were measured (Figures 3(a) and 3(b)). The results showed that chrysophanol (30 and 70 *μ*m) caused an increase in ROS production in FaDu and SAS (Figure 3(a)); however, the expression of p53 or p21 was increased in FaDu but decreased in SAS (Figure 3(b) and supplementary Figure 2). We suggest that the conflicting results are based on the different genetic backgrounds. The state of p53 in FaDu was a mutation of CGG to CTG that occurred at codon 248 [25, 26], and SAS was shown to express the wild-type p53 genotype [27, 28]. The downstream gene p21 was upregulated following p53 activation and mediated cell cycle progression [29]. Moreover, the expression of cyclinD1, CDK4, cdc2, and CDK2 was downregulated under chrysophanol (30 and 70 *μ*m) treatment in FaDu and SAS (Figure 3(c) and supplementary Figure 3). Chrysophanol also arrested the cell cycle at G1 (Figure 3(d)). To further confirm whether chrysophanol caused cell death via ROS production, the results demonstrated that NAC alleviated cell death in 70 *μ*m chrysophanol-treated FaDu and SAS (Figure 3(e)). Therefore, the data showed that chrysophanol triggered cell to death via ROS production and G1 phase arrest.

### 3.4. Chrysophanol Inhibited Cell Migration/Metastasis via an EMT-Dependent but ROS-Independent Pathway

**Figure 4:**
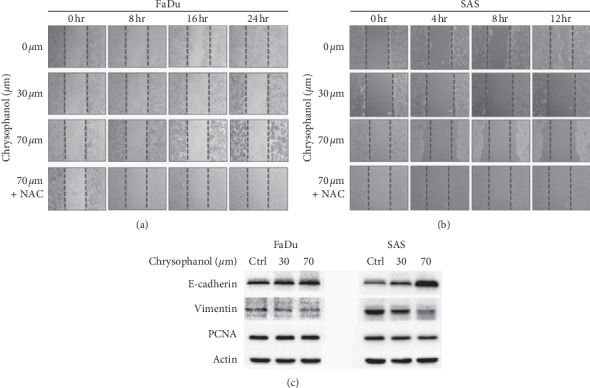
Chrysophanol inhibits cell migration and EMT. (a, b) Migration was assessed following mechanical wound healing. Cell migration was evaluated at 24 h after treatment with various doses of chrysophanol. Effect of NAC on cell migration in the presence of 70 *μ*m chrysophanol. (c) The expression of E-cadherin, vimentin, and PCNA was analyzed by western blotting in chrysophanol-treated cells (30 and 70 *μ*m) or control (Ctrl, 0 *μ*m).

Accumulating evidence demonstrates that EMT plays a critical role in oral cancer invasion, migration, and metastasis [30, 31]. To study the effect of chrysophanol on cell migration in FaDu and SAS, the wound healing assay was used. Results showed that chrysophanol strongly inhibited cell migration; however, the inhibitory effect was not reversed after NAC treatment (Figures 4(a) and 4(b)). The data demonstrated that chrysophanol has an antimigration effect via a ROS-independent pathway. Furthermore, loss of E-cadherin is a positive signal for metastasis [32], and vimentin upregulation has been reported in correlation with metastasis and may be a poor prognosis indicator in oral cancer [33]. Results showed that E-cadherin increased but vimentin decreased after 70 *μ*m chrysophanol treatment (Figure 4(c) and supplementary Figures 4(a) and 4(b)). Additionally, proliferating cell nuclear antigen (PCNA) can be identified as a proliferation marker in tumor formation [34]. Conflicting results showed that chrysophanol decreased the PCNA expression in SAS (Figure 4(c), right panel; supplementary Figure 4(c), right panel) in a dose-dependent manner, but the same effect was not observed in FaDu (Figure 4(c), left panel; supplementary Figure 4(c), left panel). Chen et al. reported an interesting finding that FaDu has increased intracellular stiffness and an epithelial-type; on the other hand, SAS has no significant difference in intracellular stiffness and a mesenchymal-type [35]. Moreover, some studies reported that the inverse correlation between intracellular stiffness, and invasiveness was revealed in oral cancer cell lines [36, 37]. To further make a conclusion that chrysophanol is a novel therapeutic agent for treating oral cancer. We used cisplatin [38] as a positive control to compare the capability of chrysophanol. Results showed that cisplatin (30–100 *μ*m) inhibited cell viability in a concentration-dependent manner (Supplementary Figure 5(a)). Interestingly, conflicting results showed E-cadherin decreased in cisplatin-treated SAS (Supplementary Figure 5(a)) but was increased chrysophanol-treated SAS (Figure 4(c), right panel; supplementary Figure 4(c), right panel). Similar results showed that the decreasing in caspase-3 and increasing in cleaved-caspase-3 were detected after cisplatin (Supplementary Figure 5(b)) and chrysophanol (Figure 2(c) and supplementary Figure 1) treatment. The data also indicated that cisplatin has antimigration effect on SAS (Supplementary Figure 5(c)). On the other hand, to elicit substantial inhibition of cell viability (Figure 1), migration is considered a more sensitive function; therefore, usually lower concentrations, subtoxic or subantiproliferative doses, are tested. Results showed that chrysophanol inhibited cell migration in a lower concentration (10 or 8 *μ*m) in both FaDu and SAS (Supplementary Figures 6, 7(a), and 7(b)). The similar results indicated that cisplatin also inhibited cell migration in a lower concentration (20 *μ*m) in SAS (Supplementary Figure 7(c)). Therefore, we suggest that chrysophanol and cisplatin may alleviate cell migration/metastasis via an EMT-dependent and -independent pathway, respectively.

**Figure 5:**
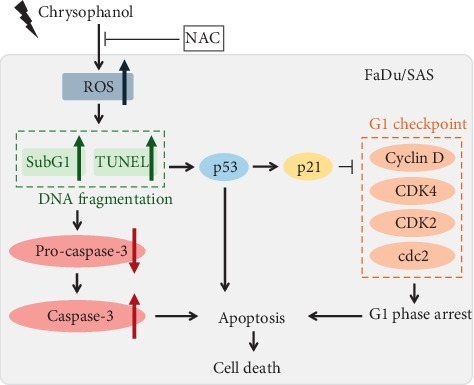
Chrysophanol caused cell death via apoptosis. Chrysophanol induced ROS production, increasing the subG1 population and phenomena of apoptosis. NAC reversed chrysophanol-induced cell cytotoxicity. On the other hand, chrysophanol caused G1 phase arrest via regulations of p53, p21, and cell cycle G1 checkpoint-associated molecules.

In conclusion, chrysophanol significantly induced cell death via apoptosis by inducing ROS accumulation. On the other hand, chrysophanol caused cell cycle arrest and inhibited migration/metastasis via an EMT-dependent but ROS-independent pathway (Figure 5). Our pharmacological findings support further development of chrysophanol as a novel therapeutic agent for treating oral cancer.

## Data Availability

The original data used to support the findings of this study are included within the article.

## Supplementary Materials

Supplementary Materials Supplementary Figure 1: quantification of western blots represented in Figure 2(c). Triplicates of the experiments shown in Figure 2(c) are quantified using densitometry (GraphPad Prism8 software). Densitometric analysis of all samples normalized against the level of total protein. The relative expression of procaspase 3 and *β*-actin are presented as procaspase 3/*β*-actin (fold) in (a) FaDu and (b) SAS, and caspase 3 and *β*-actin are presented as caspase-3/*β*-actin (fold) in (c) FaDu and (d) SAS. We used *β*-actin as the reference. All data are presented as mean ± SEM, *n* = 3. ^*∗*^*p* < 0.05. ^*∗∗*^*p* < 0.01 compared with control (Ctrl). Supplementary Figure 2: quantification of western blots represented in Figure 3B. Triplicates of the experiments shown in Figure 2C were quantified using densitometry (GraphPad Prism8 software). Densitometric analysis of all samples normalized against the level of total protein. The relative expression of p53 and *β*-actin is presented as p53/*β*-actin (fold) in (a) FaDu and (b) SAS, and p21 and *β*-actin are presented as p21/*β*-actin (fold) in (c) FaDu and (d) SAS. We used *β*-actin as the reference. All data are presented as mean ± SEM, *n* = 3. ^*∗*^*p* < 0.05. ^*∗∗*^*p* < 0.01 compared with control (Ctrl). Supplementary Figure 3: quantification of western blots represented in Figure 3C. Triplicates of the experiments shown in Figure 2C were quantified using densitometry (GraphPad Prism8 software). Densitometric analysis of all samples normalized against the level of total protein. The relative expression of cyclin D1 and *β*-actin is presented as cyclin D1/*β*-actin (fold) in (a) FaDu and SAS, and that of CDK4 and *β*-actin is presented as CDK4/*β*-actin (fold) in (b) FaDu and SAS. Densitometric analysis of all samples normalized against the level of total protein. The relative expression of cdc2 and *β*-actin is presented as cdc2/*β*-actin (fold) in (c) FaDu and SAS, and that of CDK2 and *β*-actin is presented as CDK2/*β*-actin (fold) in (d) FaDu and SAS. We used *β*-actin as the reference. All data are presented as mean ± SEM, *n* = 3. ^*∗*^*p* < 0.05. ^*∗∗*^*p* < 0.01 compared with control (Ctrl). Supplementary Figure 4: quantification of western blots represented in Figure 4C. Triplicates of the experiments shown in Figure 2C were quantified using densitometry (GraphPad Prism8 software). Densitometric analysis of all samples normalized against the level of total protein. The relative expression of E-cadherin and *β*-actin is presented as E-cadherin/*β*-actin (fold) in (a) FaDu and SAS and that of vimentin and *β*-actin is presented as vimentin/*β*-actin (fold) in (b) FaDu and SAS. Densitometric analysis of all samples normalized against the level of total protein. The relative expression of PCNA and *β*-actin is presented as PCNA/*β*-actin (fold) in (c) FaDu and SAS. All data are presented as mean ± SEM, *n* = 3. ^*∗*^*p* < 0.05 compared with control (Ctrl). Supplementary Figure 5: effect of cisplatin on cell viability, EMT, apoptosis, and migration. (a) After the incubation time course, cell viability was determined by using the MTT assay. Data are presented as mean ± SEM, *n* = 3. ^*∗*^*p* < 0.05 compared with control (Ctrl, 0 *μ*m). (b) The expression of E-cadherin, vimentin, PCNA, caspase-3, and cleaved-caspase-3 was analyzed by western blotting in cisplatin-treated cells (43.8 *μ*m) or control (Ctrl, 0 *μ*m). (c) Migration was assessed following mechanical wound healing. Cell migration was evaluated at 12 h after treatment with 43.8 *μ*m cisplatin. Supplementary Figure 6: chrysophanol inhibits cell migration in a lower concentration. Migration was assessed following mechanical wound healing in FaDu (a) and SAS (b). Cell migration was evaluated at 12 h or 24 h after treatment with 10 *μ*m of chrysophanol or not (control). Supplementary Figure 7: chrysophanol inhibits cell migration in a lower concentration than IC50. Migration was assessed following mechanical wound healing in FaDu (a) and SAS (b-c). Cell migration was evaluated at 12 h or 24 h after treatment with 8 *μ*m of chrysophanol (a-b) or 20 *μ*m of cisplatin (c). Control indicated an absence of chrysophanol or cisplatin.
