# Supplementary material for: Chrysophanol Regulates Cell Death, Metastasis, and Reactive Oxygen Species Production in Oral Cancer Cell Lines
Source: Evid Based Complement Alternat Med. 2020 May 26;2020:5867064. doi: 10.1155/2020/5867064 (PMC7271060; doi:10.1155/2020/5867064)
Supplement: Supplementary Materials — Supplementary Figure 1: quantification of western blots represented in Figure 2(c). Triplicates of the experiments shown in Figure 2(c) are quantified using densitometry (GraphPad Prism8 software). Densitometric analysis of all samples normalized against the level of total protein. The relative expression of procaspase 3 and β-actin are presented as procaspase 3/β-actin (fold) in (a) FaDu and (b) SAS, and caspase 3 and β-actin are presented as caspase-3/β-actin (fold) in (c) FaDu and (d) SAS. We used β-actin as the reference. All data are presented as mean ± SEM, n = 3. ∗p < 0.05. ∗∗p < 0.01 compared with control (Ctrl). Supplementary Figure 2: quantification of western blots represented in Figure 3B. Triplicates of the experiments shown in Figure 2C were quantified using densitometry (GraphPad Prism8 software). Densitometric analysis of all samples normalized against the level of total protein. The relative expression of p53 and β-actin is presented as p53/β-actin (fold) in (a) FaDu and (b) SAS, and p21 and β-actin are presented as p21/β-actin (fold) in (c) FaDu and (d) SAS. We used β-actin as the reference. All data are presented as mean ± SEM, n = 3. ∗p < 0.05. ∗∗p < 0.01 compared with control (Ctrl). Supplementary Figure 3: quantification of western blots represented in Figure 3C. Triplicates of the experiments shown in Figure 2C were quantified using densitometry (GraphPad Prism8 software). Densitometric analysis of all samples normalized against the level of total protein. The relative expression of cyclin D1 and β-actin is presented as cyclin D1/β-actin (fold) in (a) FaDu and SAS, and that of CDK4 and β-actin is presented as CDK4/β-actin (fold) in (b) FaDu and SAS. Densitometric analysis of all samples normalized against the level of total protein. The relative expression of cdc2 and β-actin is presented as cdc2/β-actin (fold) in (c) FaDu and SAS, and that of CDK2 and β-actin is presented as CDK2/β-actin (fold) in (d) FaDu and SAS. We used β-actin as the refe [file 5867064.f1.pdf]

Supplementary figure 1

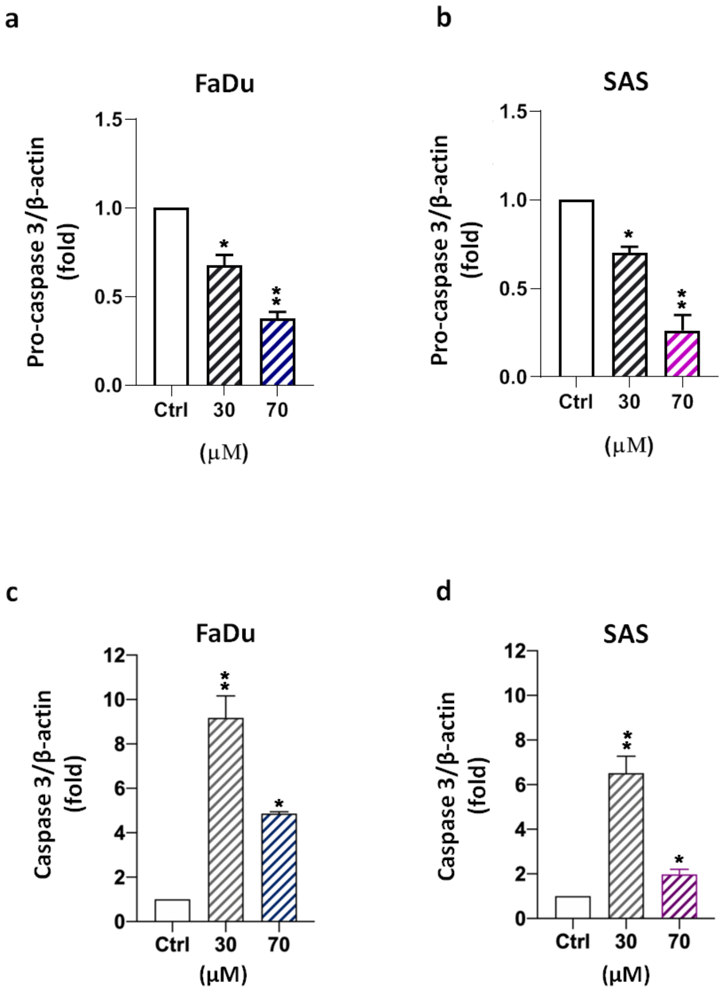

Supplementary Fig. 1 Quantification of Western blots represented in Figure 2C. Triplicates of the experiments shown in Figure 2C were quantified using densitometry (GraphPad Prism8 software). Densitometric analysis of all samples normalized against the level of total protein. The relative expression of pro-caspase 3 and β-actin are presented as pro-caspase 3/β-actin (fold) in (a) FaDu and (b) SAS, and caspase 3 and β-actin are presented as caspase-3/β-actin (fold) in (c) FaDu and (d) SAS. We used β-actin as the reference. All data are presented as means ± SEM, n = 3. *$p < 0.05$. **$p < 0.01$ compared to control (Ctrl).

Supplementary figure 2

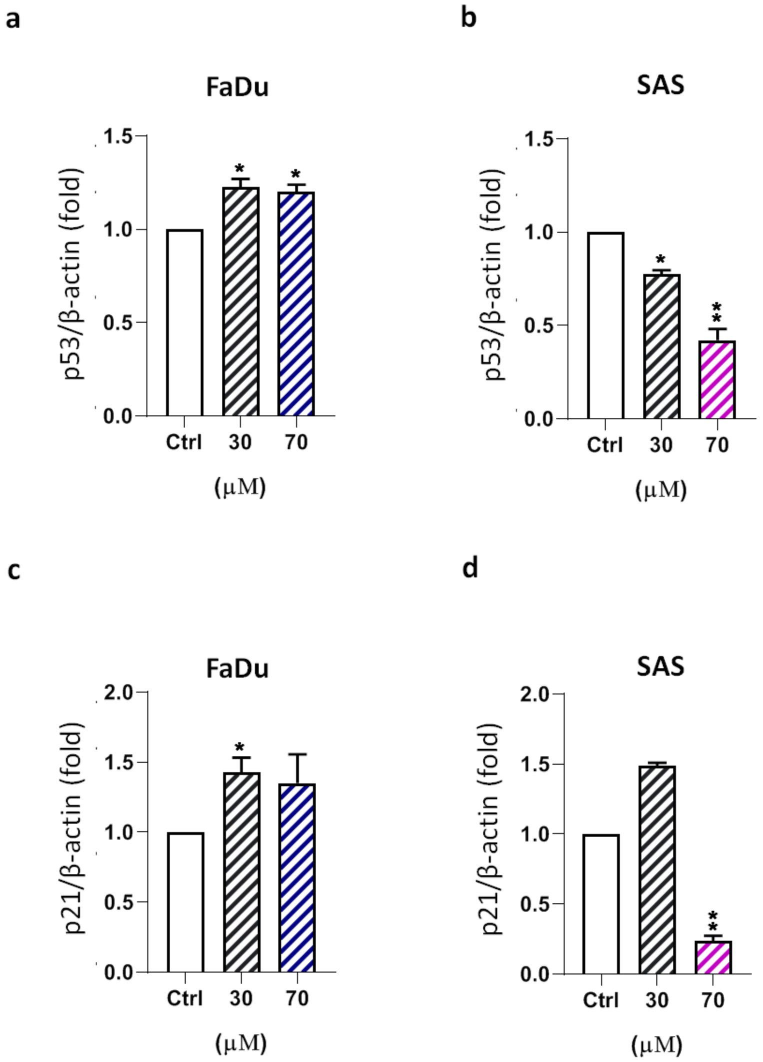

Supplementary Fig. 2 Quantification of Western blots represented in Figure 3B. Triplicates of the experiments shown in Figure 2C were quantified using densitometry (GraphPad Prism8 software). Densitometric analysis of all samples normalized against the level of total protein. The relative expression of p53 and β-actin are presented as p53/β-actin (fold) in (a) FaDu and (b) SAS, and p21 and β-actin are presented as p21/β-actin (fold) in (c) FaDu and (d) SAS. We used β-actin as the reference. All data are presented as means ± SEM, n = 3. $^*p < 0.05$. $^{**}p < 0.01$ compared to control (Ctrl).

Supplementary figure 3

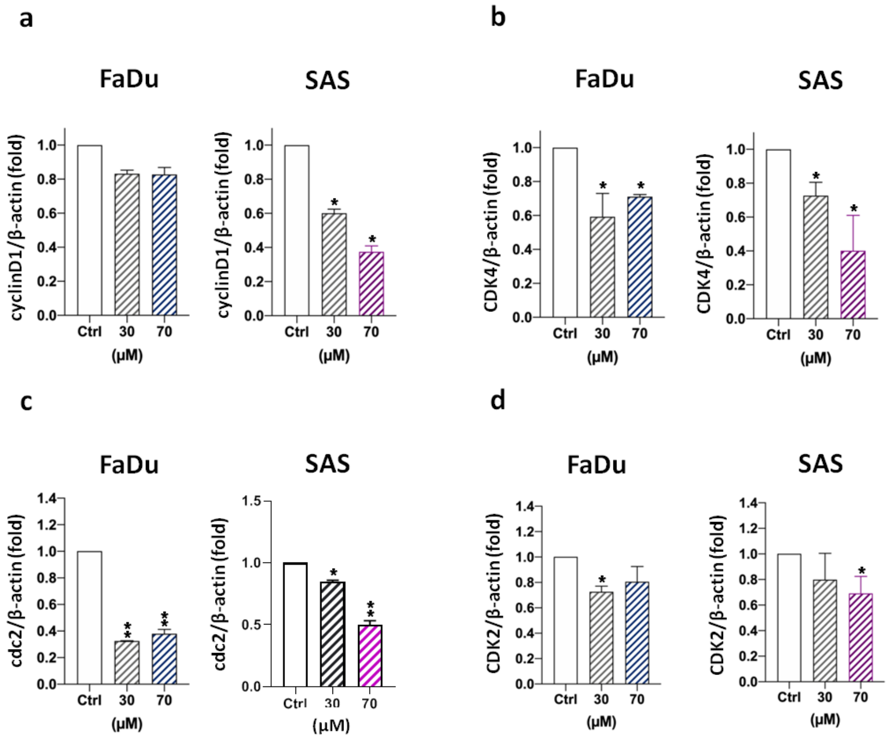

Supplementary Fig. 3 Quantification of Western blots represented in Figure 3C. Triplicates of the experiments shown in Figure 2C were quantified using densitometry (GraphPad Prism8 software). Densitometric analysis of all samples normalized against the level of total protein. The relative expression of cyclin D1 and β-actin is presented as cyclin D1/β-actin (fold) in (a) FaDu and SAS, and that of CDK4 and β-actin is presented as CDK4/β-actin (fold) in (b) FaDu and SAS. Densitometric analysis of all samples normalized against the level of total protein. The relative expression of cdc2and β-actin is presented as cdc2/β-actin (fold) in (c) FaDu and SAS, and that of CDK2 and β-actin is presented as CDK2/β-actin (fold) in (d) FaDu and SAS. We used β-actin as the reference. All data are presented as means ± SEM, n = 3. *$p < 0.05$. **$p < 0.01$ compared to control (Ctrl).

Supplementary figure 4

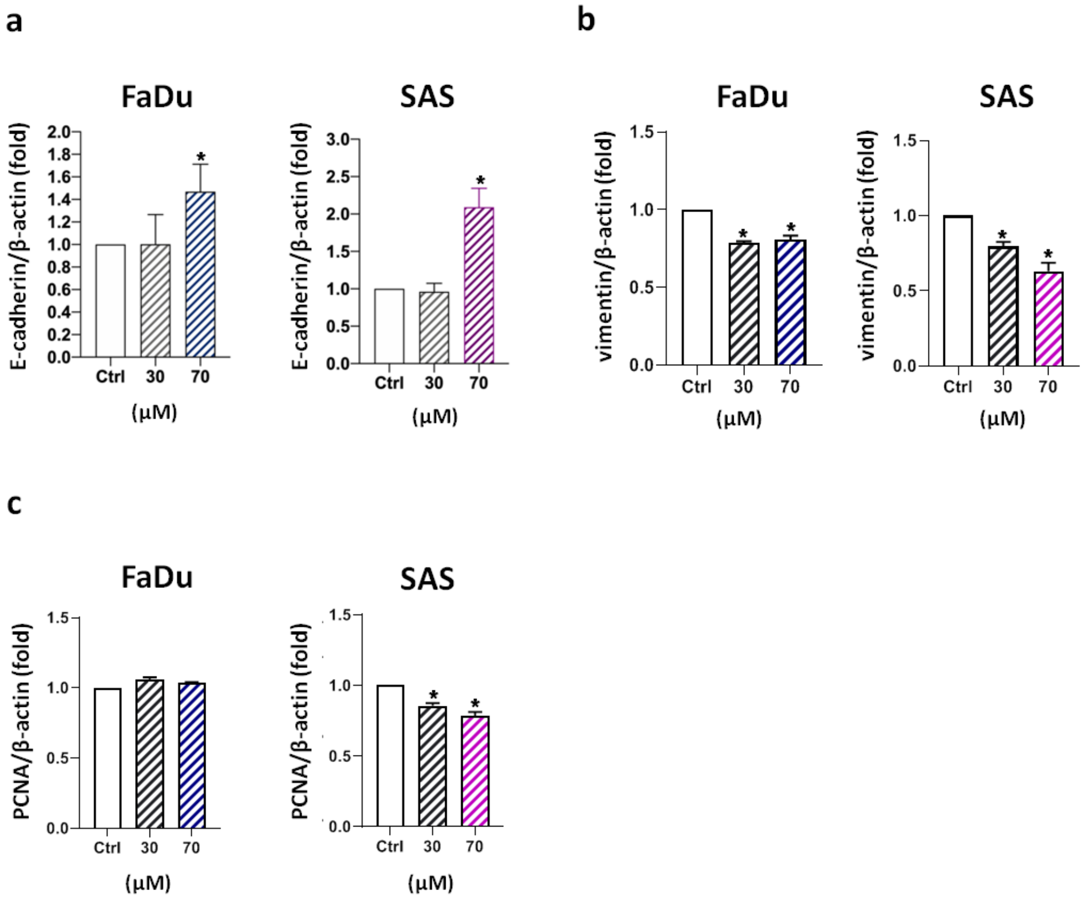

Supplementary Fig. 4 Quantification of Western blots represented in Figure 4C. Triplicates of the experiments shown in Figure 2C were quantified using densitometry (GraphPad Prism8 software). Densitometric analysis of all samples normalized against the level of total protein. The relative expression of E-cadherin and β-actin is presented as E-cadherin/β-actin (fold) in (a) FaDu and SAS, and that of vimentin and β-actin is presented as vimentin/β-actin (fold) in (b) FaDu and SAS. Densitometric analysis of all samples normalized against the level of total protein. The relative expression of PCNA and β-actin is presented as PCNA/β-actin (fold) in (c) FaDu and SAS. All data are presented as means ± SEM, n = 3. *p < 0.05 compared to control (Ctrl).

Supplementary figure 5

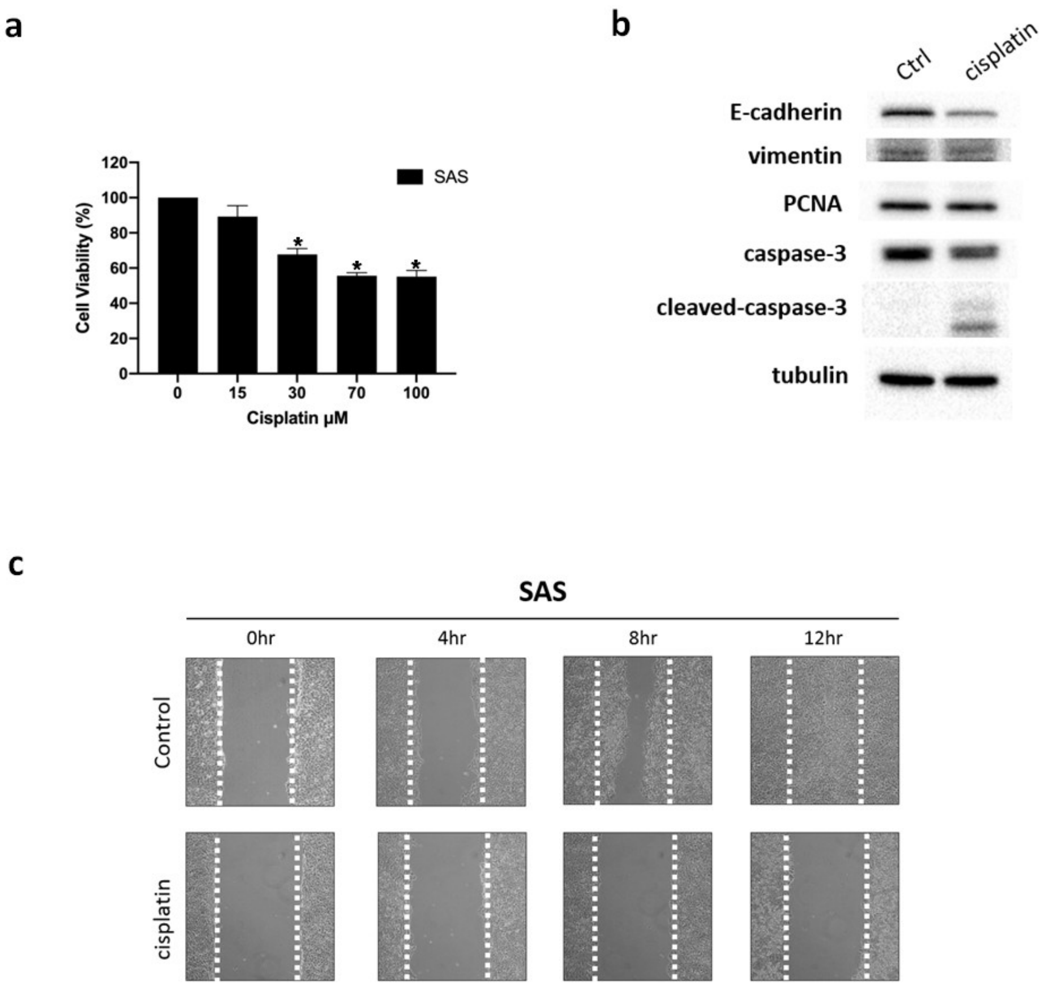

Supplementary Fig. 5 Effect of cisplatin on cell viability, EMT, apoptosis, and migration. (a) After the incubation time course, cell viability was determined by using the MTT assay. Data are presented as means ± SEM, n = 3. *$p < 0.05$ compared to control (Ctrl, 0 μM).(b) The expression of E-cadherin, vimentin, PCNA, caspase-3, and cleaved-caspase-3 was analyzed by western blotting in cisplatin-treated cells (43.8 μM) or control (Ctrl, 0 μM). (c) Migration was assessed following mechanical wound healing. Cell migration was evaluated at 12 h after treatment with 43.8μM cisplatin.

Supplementary figure 6

Supplementary Fig. 6 Chrysophanol inhibits cell migration in a lower concentration.

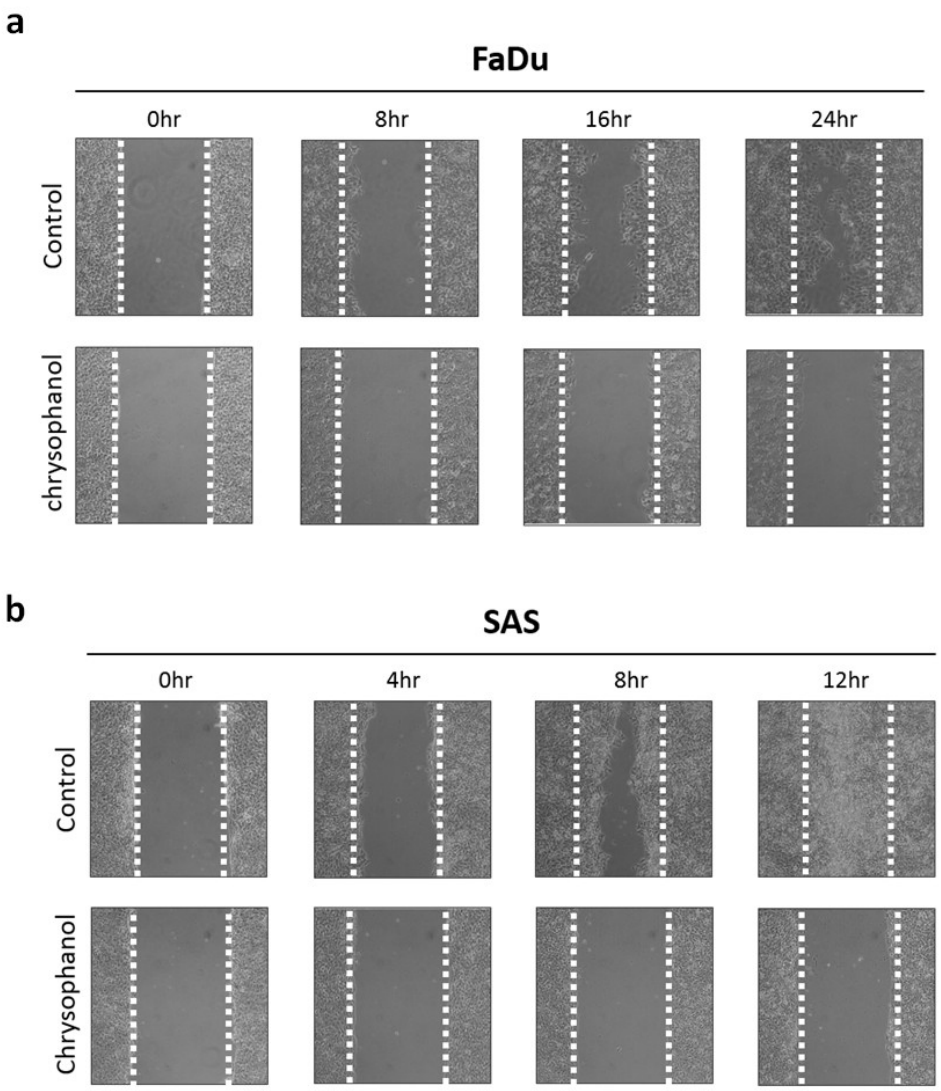

Migration was assessed following mechanical wound healing in FaDu (a) and SAS (b). Cell migration was evaluated at 12 h or 24h after treatment with 10 µM of chrysophanol or not (Control).

Supplementary figure 7

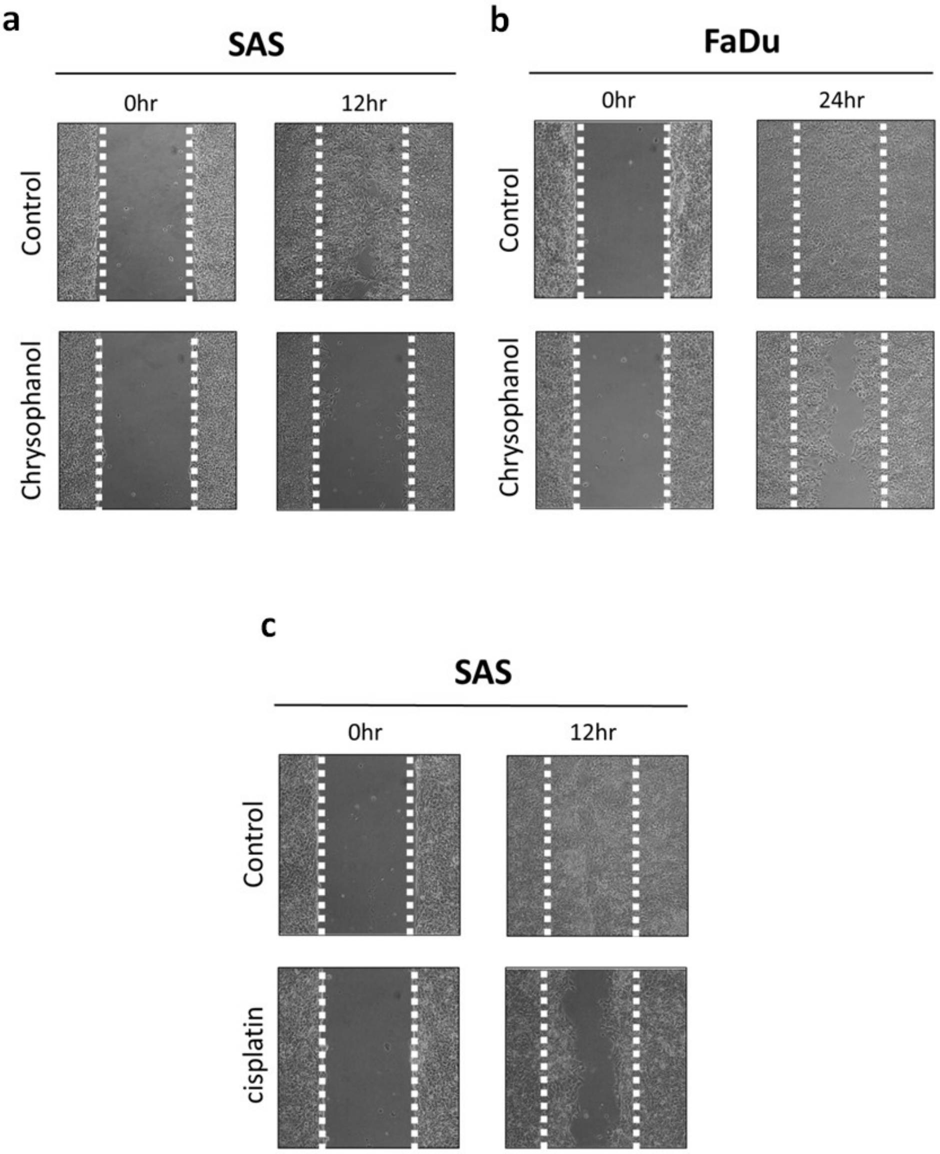

Supplementary Fig. 7 Chrysophanol inhibits cell migration in a lower concentration than IC50. Migration was assessed following mechanical wound healing in FaDu (a) and SAS (b-c). Cell migration was evaluated at 12 h or 24h after treatment with 8 µM of chrysophanol (a-b) or 20 µM of cisplatin (c). Control indicated in absence of chrysophanol or cisplatin.
